# Physical Activity Intensity Measurement and Health: State of the Art and Future Directions for Scientific Research

**DOI:** 10.3390/ijerph20116027

**Published:** 2023-06-01

**Authors:** Juan Pablo Rey-López, Dong Hoon Lee, Gerson Ferrari, Edward Giovannucci, Leandro F. M. Rezende

**Affiliations:** 1 Faculty of Health Sciences, Universidad International de Valencia (VIU), 46002 Valencia, Spain; 2 Faculty of Sports, Universidad Católica de Murcia (UCAM), 30107 Murcia, Spain; 3 Department of Sport Industry Studies, Yonsei University, Seoul 03722, Republic of Korea; 4 Department of Nutrition, Harvard T.H. Chan School of Public Health, Boston, MA 02115, USA; 5 Escuela de Ciencias de la Actividad Física, el Deporte y la Salud, Universidad de Santiago de Chile (USACH), Santiago 9170022, Chile; 6 Facultad de Ciencias de la Salud, Universidad Autónoma de Chile, Providencia 7500912, Chile; 7 Department of Epidemiology, Harvard T.H. Chan School of Public Health, Boston, MA 02115, USA; 8 Department of Medicine, Channing Division of Network Medicine, Brigham and Women’s Hospital and Harvard Medical School, Boston, MA 02115, USA; 9 Department of Preventive Medicine, Escola Paulista de Medicina, Universidade Federal de Sao Paulo, Sao Paulo 04023-900, Brazil

**Keywords:** reliability, mortality, wrist accelerometers, criterion validity

## Abstract

Physical activity guidelines for health recommend any type of unstructured physical activity for health promotion. Adults should perform at least 150–300 min per week of moderate intensity or 75–150 min per week of vigorous intensity activities, or an equivalent combination of the two intensities. However, the relationship between physical activity intensity and longevity remains a debated topic, with conflicting perspectives offered by epidemiologists, clinical exercise physiologists or anthropologists. This paper addresses the current known role of physical activity intensity (in particular vigorous versus moderate intensity) on mortality and the existing problems of measurement. Given the diversity of existing proposals to categorize physical activity intensity, we call for a common methodology. Device-based physical activity measurements (e.g., wrist accelerometers) have been proposed as a valid method to measure physical activity intensity. An appraisal of the results reported in the literature, however, highlights that wrist accelerometers have not yet demonstrated sufficient criterion validity when they are compared to indirect calorimetry. Novel biosensors and wrist accelerometers will help us understand how different metrics of physical activity relates to human health, however, all these technologies are not enough mature to provide personalized applications for healthcare or sports performance.

## 1. Introduction

Physical activity is associated with numerous health benefits for humans. Nonetheless, compelling evidence from evolutionary biology and anthropology indicates that many attitudes related to exercise are plagued by misconceptions rather than robust, independent and accurate scientific knowledge [1]. A common example of myth is that “we are born to run” [1]. Indeed, “our bodies were selected to spend enough but not too much energy on non-reproductive functions, including physical activity” [1]. In prehistoric times, food was a precious resource for our ancestors, and therefore allocating too much energy in physical activity had trade-offs: a lower energy allocation for reproductive, growing or maintenance purposes [1]. Therefore, the amount of activity accumulated by today’s elite-level endurance athletes may not necessarily be optimal for health promotion purposes as they exceed the volume and predominant intensity that our species did in the past [2].

Health promotion encompasses actions at individual level, and physical activity remains a cornerstone in primary and secondary prevention. The first-ever physical activity guidelines for health were based on doing vigorous aerobic exercise [3]. Later, the guidelines became more public health oriented including any type of unstructured physical activity intensity [3]. According to the latest updated guidelines by the U.S Department of Health and Human Services (2018) [4] and the World Health Organization (2020) [5], adults should perform around 150–300 min per week of moderate intensity, or 75–150 min per week of vigorous intensity activities, or an equivalent combination of the two intensities (MVPA), where one minute of vigorous is equivalent to two minutes of moderate. Evidence indicates that compared with a physically inactive lifestyle, physical activity volume is inversely associated with mortality [4]. The relationship between physical activity intensity and mortality has not been settled, with conflicting results found by different epidemiologists [6]. Indeed, a person or a population may meet physical activity recommendations for health using a huge diversity of combinations of moderate or vigorous physical activity intensities. In some cohort studies, and for the same amount of moderate to vigorous physical activity, the group with the greatest proportion of vigorous activities (compared with none vigorous or a very low amount vigorous) had a lower risk of all-cause mortality. However, no differences in mortality were found in other cohort studies [6]. The latter would imply that accumulate enough volume of physical activities at moderate intensity would be sufficient to reduce mortality risk.

Questionnaires are a convenient tool to describe participants’ physical activity levels in large epidemiological studies [6], but are limited in the sense that they may introduce measurement errors. Device-based physical activity measurements (wrist accelerometers) have become very popular among consumers and researchers, but wrist accelerometers’ lack of accuracy in collecting certain types of physical activities and their main metrics (intensity, and energy expenditure) still require validation against the gold standards of measurement.

Epidemiologists have historically employed questionnaires to describe physical activity (type of activities, duration, frequency and intensity). Nevertheless, today accelerometers are progressively replacing (or complementing) questionnaires in scientific studies due to their advantages. Light intensity activities and other new emerging metrics of physical activity, such as fragmented physical activity, sleep time or gait parameters, have now become measurable [7,8,9].

Physical activity intensity of participants recruited from large epidemiological studies has been expressed in an absolute scale (by metabolic equivalent of tasks, METs) because of its simplicity and low cost. Moderate intensities are those activities that produce an energy expenditure ranging from 3 to 5.9 METs, and vigorous intensities are those eliciting ≥6 METs. However, absolute measures may result in misclassification of intensity because they do not take into account body weight, sex and fitness level [10]. Relative intensity takes into account a person’s cardiorespiratory fitness or how hard the person feels he or she is exercising (for example, on a 0 to 10 scale). Methods to define relative physical activity intensity rely on the determination of percentages (%) of maximal oxygen consumption (VO2 max) or oxygen uptake reserve (VO2 R) (Table 1). Accurate assessments of relative physical activity intensity are, however, more expensive and time consuming for researchers and participants because they must be evaluated by well-trained personnel in a laboratory. Other physiological variables (maximum heart rate, heart rate reserve, ventilatory or lactate thresholds) may also be used to identify intensity zones [10].

As shown in Table 1, there are a variety of cut-points to classify physical activity intensity levels (by VO_2_ max or VO_2_ R) by different scientific societies [4,10,11,12] and researchers (Table 1) [13,14,15]. To illustrate with one example how misleading it can be to categorize physical activity intensity, let us imagine an elite endurance athlete (VO_2_ max: 70 mL/kg/min) who runs 75 min at 79% of their maximal VO_2_. In an absolute intensity scale, this athlete is doing vigorous activity (≥6 METs) and meeting with physical activity guidelines. This intensity level, however, would be classified as low intensity among coaches of world-class endurance athletes [15] or moderate intensity [13], hard intensity [12], or vigorous intensity [11] or very vigorous intensity [14] for others.

Data in Table 1 indicate that the research community must urgently adopt the same terminology (and cut-points) to categorize relative physical activity intensity levels. Moreover, consensus must exist on the physiological marker (and method of measurement) that researchers should rely to define relative physical activity intensity levels.

Compared with fixed percentages of VO_2_ max or VO_2_ R, we argue that information of lactate thresholds might be a more suitable methodology for novel therapeutical avenues, in addition to its role in guiding sport performance [15]. Lactate, considered for long time a waste product of metabolism associated with fatigue, is currently gaining much attention among healthcare professionals because it plays a significant role in the regulation of energy metabolism, immunity responses, memory formation, wound healing, and tumour development [16]. Nonetheless, the use of blood lactate to support decision-making in healthcare or sports settings has not arrived yet, as the science has only reached an early stage (research and development) of the technology life-cycle. For example, in a recent study in which five participants carried out 30 min of aerobic exercise, authors reported a good agreement between venous lactate levels and interstitial lactate levels using a minimally invasive microneedle patch. However, within individuals’ differences between the two methods were large (95% CI difference of ±1.89 mmol/L) [17]. Another barrier is how informative lactate biosensors are placed in different anatomical locations. Fingertip blood lactate concentrations are higher than venous blood during incremental or submaximal exercise protocols in humans [18].

In this opinion article, we argue that wrist accelerometers still have insufficient criterion validity compared to when laboratory studies have measured intensity by indirect calorimetry. Moreover, we briefly discuss the main findings of epidemiological studies that compared mortality rates of participants with different proportions of vigorous activity (reference group: no vigorous or the lowest proportion of vigorous). Finally, we report the main findings of the first clinical trial published (in older adults) evaluating the impact on mortality of a supervised program of vigorous intensity (versus another group of moderate intensity).

## 2. Methods

Our selection of research articles was based on studies identified in a recent review of Liu et al. [9], where the authors examined the criterion validity of wrist accelerometers versus measurements of total physical activity energy expenditure (using doubly labelled water) and physical activity energy expenditure of some activities evaluated in laboratories (indirect calorimetry). To discuss the role of vigorous and moderate intensity on mortality, we cite the main references in this field according to our knowledge after working with large cohort epidemiological studies of physical activity and health.

## 3. Results and Discussion

### 3.1. Are Wrist Accelerometers Valid Tools to Evaluate Physical Activity Intensity?

A growing number of observational and randomized clinical trials are incorporating accelerometers to measure physical activity. Accelerometers are small devices generally worn at the hip or wrist, although the latter location is gaining popularity among physical activity epidemiologists due to their lower participant burden and higher compliance [9]. In fact, several eminent epidemiological projects have measured physical activity through wrist accelerometers (e.g., the UK Biobank, NHANES, Whitehall II, and the Pelotas birth cohorts) [9]. Consequently, we decided to discuss hereafter the validity of wrist accelerometers to assess physical activity intensity or physical activity energy expenditure (PAEE). Recently, Liu et al. [9] reviewed studies that included wrist accelerometers and compared with the gold standards of PAEE or a reference method of physical activity intensity (calorimetry). Regarding physical activity intensity, Liu et al. identified eight studies that established intensity acceleration cut-points using calorimetry, and concluded (without any detailed justification) that “wrist-accelerometers are valid instruments of classifying physical activity intensities”. Contrary to Liu et al.’s interpretation, we argue that the results of validation studies of wrist accelerometers still need to demonstrate an acceptable criterion of validity performance to classify intensity. To assess criterion validity, intensity scores obtained with accelerometers (counts) would require comparison with those obtained with the gold standards (values of oxygen consumption or other physiological markers). Both variables, counts and oxygen consumption, must be understood as continuous variable for analytical purposes. As their units of measurement are different (accelerations and oxygen values), a suitable statistic test of criterion validity is Spearman’s or Pearson’s coefficient correlations [19]. Table 2 shows coefficient correlations of seven validation [20,21,22,23,24,25,26] studies identified by Liu et al. [9] (Note: one validation study was excluded from our opinion article [27] as the commercial distribution of the accelerometer (Actical, Phillips Respironics, Bend, OR, USA) has been discontinued).

Remarkably, only two validation studies [20,21] found Spearman’s or Pearson’s coefficient correlations above 0.7, which is considered the acceptable correlation level threshold of criterion validity. Three out of the seven validation studies did not even report coefficient correlations in their validation analyses [22,23,24]. Instead, authors reported other statistical parameters, such as sensitivity and specificity values. The latter statistics may be less informative to study criterion-validity because they are indicated to compare dichotomous variables, with the same units of measurement [19]. Another important limitation is that all validation studies expressed physical activity intensity in an absolute scale, which is more likely to produce individual misclassification bias. To our knowledge, only one study in the literature has evaluated the validity of a wrist accelerometer (GENEA) using a relative intensity physical activity scale. In 98 recreational runners (30–45 years, both sexes), Hernando et al. [14] found high correlation Spearman values (0.886, *p*-value = 2.20 × 10^−16^) between raw accelerations and percentages of VO2 max estimated through a running test in a treadmill. This finding must, however, be interpreted with caution because authors did not mimic habitual physical activities of participants in free-living conditions. Indeed, one researcher validates not a measurement instrument, but rather some uses of the instrument [19]. In this sense, wrist accelerometers still have substantial technical limitations to capture certain physical activities (e.g., strength training movements, swimming and cycling). For all the reasons mentioned above, it seems misleading to conclude that current wrist accelerometers can accurately measure physical activity intensity.

### 3.2. Are Wrist Accelerometers (for Researchers) or Commercial Wearables (for Consumers) Able to Assess Physical Activity Energy Expenditure (PAEE)?

Another existing barrier of current accelerometers is their inability to accurately estimate PAEE measurements. The gold standard for measuring PAEE in free-living conditions is doubly labelled water (DLW) [28]. Procedures of DLW are complex and expensive as they require the collection of urine samples and measures of resting metabolic rate [28]. Unsurprisingly, a low number of studies in humans (with small samples) have incorporated DLW to validate physical activity measurements. A recent systematic review concluded that current commercial wearables are inaccurate tools to measure PAEE [29]. Regarding wrist accelerometers, very few studies have compared their validity using DLW [9]. In Liu et al.’s [9] review, only two studies [30,31] compared physical activity using wrist accelerometers with DLW, showing moderate intraclass correlations values (0.61–0.68) in adults in free-living conditions.

Of note, acceleration only explained 19% of the variance of physical activity energy expenditure in a sample of pregnant and non-pregnant women [31].

### 3.3. Physical Activity Intensity and Mortality: Beyond Measurement Errors

As we have discussed in the previous paragraphs, classifying individuals in different intensity levels still require the development of more accurate technologies and a common consensus among researchers on how to categorize indexes of relative intensity. However, in this section, and based on our experience with cohort studies, we summarize additional obstacles (study design and analytical problems) that are impeding further scientific progress in this area. Causal inference from cohort studies must be understood as a way to emulate a hypothetical (ideal) randomized controlled trial (RCT) [32]. For the question “Does vigorous intensity physical activity provides larger mortality benefits than moderate intensity?”, a hypothetical RCT should randomly assign a vigorous activity program for the intervention group and moderate activity for controls. Each group should do the same volume of physical activity (MET hours) and mortality rates should be compared after a long follow-up (ideally with 100% adherence and no loss to follow-up).

Recently, we found that many cohort studies focused on questionnaire-based physical activity intensity and mortality [6] were at high risk of bias or possible misinterpretation because of the following reasons:

- Physical activity intensity was self-reported in questionnaires.
- A single measurement of physical activity at baseline was used in all identified studies. We have previously showed that a single baseline measurement may underestimate the benefits of physical activity on mortality [33]. Using repeated measures (cumulative average physical activity) and avoiding short lag times (e.g., excluding participants who died during the first 2 years of follow-up) may reduce measurement error and reverse causation, respectively.
- In some studies, authors included an inadequate comparator group in their analyses (i.e., physically inactive rather than physically active individuals at moderate intensity) when the aim was to examine the additional mortality benefits of doing more intense versus moderate activities.
- The endpoint used in some studies was a composite variable measuring both cardiovascular disease (CVD) incidence and mortality.
- The influence of pre-existing diseases (for example, cardiometabolic diseases at baseline) was not optimally accounted for in some large epidemiological studies predisposing the studies to confounding.

The few cohort studies that have compared how different proportions of vigorous intensity versus moderate intensity (but non-vigorous) are associated with all-cause mortality were included in a meta-analysis [6]. The main conclusion of this meta-analysis was that performing the highest proportion of vigorous activity (versus moderate but non-vigorous) did not add additional protection on all-cause mortality. More recently, this conclusion has been challenged (higher duration of vigorous activity adds additional reductions on all-cause mortality) [34], but at the same time it also received validation from another large epidemiological study [35] (in separate analyses of intensity meeting physical activity recommendations (versus inactivity) based on either moderate or vigorous activity was associated with a lower all-cause mortality risk: 0.81 (95% CI, 0.76–0.87) and 0.79 (95% CI, 0.76–0.82, respectively). When we examined the joint analyses of moderate and vigorous activity in the same populations, we found that performing vigorous activity did not add additional protection when enough moderate activity was accumulated. However, all these large epidemiological studies relied on the subjective responses of individuals to classify the intensity of the physical activity. Although new epidemiological studies are incorporating wrist accelerometers (Axivity AX3, Axivity, Newcastle, UK) to classify vigorous, moderate, or light intensity activity using a machine learning scheme [36], the validity (compared with body cameras) of this accelerometer (accuracy, weighted kappa, and intraclass correlation values) for each intensity level was not reported by authors.

RCTs of physical activity intensity and mortality outcomes are, unfortunately, almost non-existent due to the high cost and difficulties associated with conducting a well-designed, long duration trial. In the first-ever published RCT in older adults (and after 5 years of follow-up), Stensvold et al. [37] found suggestive evidence of lower risk of all-cause mortality in participants allocated to high intensity interval training (n = 400) versus the group of moderate intensity (n = 387) (HR = 0.51, 95% CI, 0.25–1.02). However, some limitations of this trial must be acknowledged. Authors reported a low exercise adherence in all participants (24.8% of overall drop-outs) and a large proportion of individuals in the moderate intensity group failed to exercise at moderate intensity (see Supplementary Table 3 in reference [37]). Intention-to-treat analyses (less susceptible to confounding and more relevant for policymakers) may have introduced substantial bias due to non-adherence to the treatment [38]. “Per protocol” analyses evaluating the effect of adhering to the intervention, on the other hand, could be additionally informative, although results could reflect reverse causation (those unable to exercise having higher mortality) and confounding.

## 4. Conclusions

To advance scientific knowledge, future epidemiological studies on physical activity and mortality must incorporate much more sophisticated measurements of physical activity intensity. The integration of several technologies (wrist accelerometers or small biosensors able to quantify metabolic changes during exercise) will help us unravel how different metrics of physical activity relates to human health in the coming years.

Understanding how physical activity intensity impacts health and lower mortality rates in adulthood remains a difficult scientific endeavour. Importantly, researchers must agree on a common and most accurate device to measure physical activity intensity, and they need to pay more attention to study design and analytical issues that are impeding a more clear understanding of how physical activity intensity impacts human health.

## Figures and Tables

**Table 1:** Indexes of relative physical activity intensity suggested by different authors and scientific societies.

Author	Society	Indexes of Intensity-%VO_2_ Max			
Pellicia et al. 2021 [11]	European Society of Cardiology	Low	Moderate	Vigorous	Very high
		<40%	40–69%	70–85%	>85%
Strath et al. 2013 [12]	American Heart Association	Light	Moderate	Hard	Very hard
		25–44%	45–59%	60–84%	≥85%
Sabag et al. 2022 [13]		Light	Moderate	HIIT	SIT
		<45%	45–79%	80–100%	>100%
Hernando et al. 2018 [14]		Light	Moderate	Vigorous	Very vigorous
		≥10–24%	≥25–44%	≥45–64%	≥65–84%
Haugen et al. 2022 [15]		LIT	MIT	HIT	
		55–79	80–84%	≥85%	
		**Indexes of Intensity-%VO_2_ Reserve**			
2017 [10]	American College of Sports Medicine		Moderate	Vigorous	
			40–59%	60–89%	
2018 [4]	US Department of Health and Human Services		40–59%	60–84%	

Values are percentages of maximal oxygen consumption (%VO_2_ max) or percentages of oxygen uptake reserve (%VO_2_ R). HIIT: High intensity interval training. SIT: Sprint interval training. LIT: Low intensity training. MIT: Moderate intensity training. HIT: High intensity training.

**Table 2:** Validation studies on physical activity intensity using wrist devices accelerations and compared with calorimetry as criterion method.

Author	Sample and Protocol Used	Wrist Device (Axis, Frequency, Epoch, and Placement)	Statistical Parameters
Neil-Sztramko et al. 2017 [25]	Female (n = 30), mean age (sd): 40 (14.9), mean BMI (sd): 22.4 (3.1). Treadmill walk/run at 2.0 mph, 3.0–3.5 mph and fast self-selected speed, self-paced indoor walk at slow, medium and fast speed, stair ascend and descend, and lift and carry task.	Actiwatch 2 (uniaxial, unspecified, 15 s, non-dominant wrist)	Pearson’s correlation coefficients (r = 0.69) between counts and oxygen consumption. VPA: Sensitivity (60.4%) and specificity (81%). MPA: Sensitivity (76.8%) and specificity (77.3%).
Lee et al. 2019 [20]	Male (n = 12) Female (n = 15), age range (18–26), mean BMI (sd): 21.9 (3.2). Treadmill run at 4, 6, 8, 10 and 12 km/h.	Actiwatch 2 (uniaxial, unspecified, 1 min, both wrists) ActiGraph GT3X+ (triaxial, unspecified, 1 min, both wrists)	Spearman’s correlation coefficients: right wrist (r = 0.73), left wrist (r = 0.72) and oxygen consumption. VPA: Sensitivity was lower than 62% in both wrists and both brands. Specificity was higher than 89.6% in both wrists and both brands. MPA: Sensitivity was lower than 80% in both wrists and both brands. Specificity was higher than 76.4% in both wrists and both brands.
Rhudy et al. 2020 [24]	Male (n = 27) Female (n = 17), mean age (sd): 26.1 (9.6), mean BMI (sd): 26.1 (4.1). Each participant completed a four-stage treadmill protocol at 1.9, 3, 4, and 5.2 mph.	ActiGraph GT9X Link (triaxial, unspecified, 1 min, left wrists)	Pearson’s or Spearman’s correlation coefficients of counts with oxygen consumption were not calculated. VPA: Sensitivity and specificity higher than 90%. MPA: Sensitivity and specificity higher than 80%.
Hildebrand et al. 2014 [22]	Adults and Children: Male (n = 29) Female (n = 31), mean age (sd): Adults 34.2 (10.7), Children 8.9 (0.9). Lab-based activities including lying supine position, sitting, standing, taking off shoes standing, moving 8 items in a bookshelf, writing a sentence, putting a paper in an envelope, sitting down, treadmill walk/run at 3, 5, and 8 km/h, and stepping.	ActiGraph GT3X+ (triaxial, 60 Hz, 1 s, unspecified)	Pearson’s or Spearman’s correlation coefficients of counts with oxygen consumption were not calculated. Authors reported a classification accuracy of only 16% for moderate intensity in adults for slow walking activities (3 km/h). No values reported for vigorous. The values (0–100) indicate the accuracy expressed in percentages for the regression models compared with the true intensity measured with indirect calorimetry (i.e., 0 means that no individuals at this intensity were correctly classified by the regression model, whereas 100 means that all individuals were correctly classified).
Duncan et al. 2019 [26]	Male (n = 9) Female (n = 14), mean age (sd): 63.2 (6.5), mean BMI (sd): 26.2 (4.0). Lab-based activities including lying supine, seated reading, slow walking, medium walking, fast walking, folding laundry, sweeping the floor and cycling.	GENEActiv (triaxial, 80 Hz, 1 s, both wrists)	Pearson’s correlation coefficients: non-dominant wrist (r = 0.26); dominant wrist (r = 0.27) between counts (using data for one second epoch in 3 min activity period per activity) and oxygen consumption. Authors did not provide data of sensitivity and specificity for either MPA or VPA.
Esliger et al. 2011 [21]	Male (n = 23) Female (n = 37), mean range: 40–63 Lab-based activities including lateral recumbent, seated computer work, standing, window washing, washing dishes, shelf stacking, sweeping, treadmill walk/run at 4, 5, 6, 8, 10, and 12 km/h, stair ascent/descent at 80 steps per minute, and brisk and medium free-living walk.	GENEActiv (triaxial, 80 Hz, 1 min, both wrists)	Pearson’s correlation coefficients: left wrist (r = 0.86), right wrist (r = 0.83) between counts (using data for one minute epoch in 4 min activity period) and oxygen consumption. VPA: Sensitivity lower than 80% in both wrists. Specificity larger than 96% in both wrists. MPA: Sensitivity larger than 94% in both wrists. Specificity lower than 73% in both wrists.
Landry et al. 2015 [23]	Male and female (n = 23), mean age (sd): 70.0 (6.6), mean BMI (sd): 26.6 (5.2). Six different activities designed to mimic activities of daily living: (1) treadmill walking at four different paces; (2) sitting in a chair; (3) cleaning; (4) resistance training; (5) lying down; and (6) standing. To more closely mimic free-living activity, participants were allowed to move their arms freely during these activities.	MotionWatch 8 (triaxial, unspecified, 1 min, non-dominant wrist)	Pearson’s or Spearman’s correlation coefficients of counts with oxygen consumption were not calculated. Authors did not provide data of sensitivity and specificity for either MPA or VPA. Sensitivity lower than 35% for MVPA.

MPA: Moderate physical activity, VPA: Vigorous physical activity, MVPA: Moderate to vigorous physical activity, sd: Standard deviation, BMI: Body mass index, Hz: Hertz.

## Data Availability

Not applicable. Data are available in each original study.
